# Sex-specific reference values for total, central, and peripheral latency of motor evoked potentials from a large cohort

**DOI:** 10.3389/fnhum.2023.1152204

**Published:** 2023-06-09

**Authors:** Mariagiovanna Cantone, Giuseppe Lanza, Francesco Fisicaro, Rita Bella, Raffaele Ferri, Giovanni Pennisi, Gunnar Waterstraat, Manuela Pennisi

**Affiliations:** ^1^Neurology Unit, Policlinico University Hospital “G. Rodolico-San Marco”, Catania, Italy; ^2^Department of Surgery and Medical-Surgery Specialties, University of Catania, Catania, Italy; ^3^Clinical Neurophysiology Research Unit, Oasi Research Institute-IRCCS, Troina, Italy; ^4^Department of Biomedical and Biotechnological Sciences, University of Catania, Catania, Italy; ^5^Department of Medical and Surgical Sciences and Advanced Technologies, University of Catania, Catania, Italy; ^6^Department of Neurology and Experimental Neurology, Charité – Universitätsmedizin Berlin, Corporate Member of Freie Universität Berlin and Humboldt-Universität zu Berlin, Berlin, Germany

**Keywords:** central motor conduction time, motor evoked potentials, reference values, transcranial magnetic stimulation, sex, physical variables, translational clinical neurophysiology

## Abstract

**Background:**

Differentiating between physiologic and altered motor evoked potentials (MEPs) to transcranial magnetic stimulation (TMS) is crucial in clinical practice. Some physical characteristics, such as height and age, introduce sources of variability unrelated to neural dysfunction. We provided new age- and height-adjusted normal values for cortical latency, central motor conduction time (CMCT), and peripheral motor conduction time (PMCT) from a large cohort of healthy subjects.

**Methods:**

Previously reported data from 587 participants were re-analyzed. Nervous system disorders were ruled out by clinical examination and magnetic resonance imaging. MEP latency was determined as stimulus-to-response latency through stimulation with a circular coil over the “hot spot” of the First Dorsal Interosseous and Tibialis Anterior muscles, during mild tonic contraction. CMCT was estimated as the difference between MEP cortical latency and PMCT by radicular magnetic stimulation. Additionally, right-to-left differences were calculated. For each parameter, multiple linear regression models of increasing complexity were fitted using height, age, and sex as regressors.

**Results:**

Motor evoked potential cortical latency, PMCT, and CMCT were shown to be age- and height-dependent, although age had only a small effect on CMCT. Relying on Bayesian information criterion for model selection, MEP cortical latency and PMCT were explained best by linear models indicating a positive correlation with both height and age. Also, CMCT to lower limbs positively correlated with height and age. CMCT to upper limbs positively correlated to height, but slightly inversely correlated to age, as supported by non-parametric bootstrap analysis. Males had longer cortical latencies and CMCT to lower limbs, as well as longer PMCT and cortical latencies to upper limbs, even when accounting for differences in body height. Right-to-left-differences were independent of height, age, and sex. Based on the selected regression models, sex-specific reference values were obtained for all TMS-related latencies and inter-side differences, with adjustments for height and age, where warranted.

**Conclusion:**

A significant relationship was observed between height and age and all MEP latency values, in both upper and lower limbs. These set of reference values facilitate the evaluation of MEPs in clinical studies and research settings. Unlike previous reports, we also highlighted the contribution of sex.

## 1. Introduction

Transcranial magnetic stimulation (TMS) is commonly used in clinical practice to non-invasively evaluate the excitability of the primary motor cortex (M1) and the conduction of neural impulses along the cortico-spinal tract. Moreover, the analysis of motor evoked potentials (MEPs) has been used in the assessment of synaptic plasticity and network connectivity, both in normal subjects and in patients with several neurological and psychiatric disorders (Bella et al., 2013; Lanza et al., 2019, 2020a,b; Cantone et al., 2020, 2021; Fisicaro et al., 2020), including systemic diseases involving the central nervous system (CNS) (Pennisi et al., 2017; Lanza et al., 2018). As an example, MEP can detect involvement of the pyramidal tract in cervical spondylosis myelopathy (CSM), even in case of absent clinical signs of pyramidal tract dysfunction (Lanza et al., 2020b). Further, MEP abnormalities correlate with disability in Multiple Sclerosis (MS) (Gagliardo et al., 2007; Kale et al., 2009) and might detect MS even in the stage of clinically isolated syndrome (Rico et al., 2009). Additionally, MEPs are helpful also in objectifying subtle upper motor neuron involvement in amyotrophic lateral sclerosis (ALS) (Triggs et al., 1999; de Carvalho et al., 2003). In the study of the peripheral nervous system, MEPs can stimulate otherwise hardly accessible nerve segments, such as the proximal pudendal nerve (Shafik, 2001) or intracranial part of the facial nerve (Rimpiläinen et al., 1992). A review on the diagnostic utility of TMS has been recently provided (Vucic et al., 2023).

Briefly, TMS produces a rapid high-intensity pulse which passes unattenuated through the scalp (Hallett, 2007; Rossini and Rossi, 2007). When TMS is applied over M1, the cortex is activated through electromagnetic induction, and the impulses are transmitted along the cortico-spinal tract and peripheral nerves, so that a MEP can be recorded from a skeletal muscle, contralateral to the stimulated hemisphere, using standard electromyography (EMG) surface electrodes. Translationally, MEPs provide a direct, objective, and painless assessment of the motor system (Hallett, 1996), including the excitability of excitatory and inhibitory circuits, the integrity of central conduction pathways, and the functioning of transcallosal connections of motor cortices (Lanza et al., 2013).

Differentiating between physiologic and altered MEP responses and attributing these alterations to central or peripheral nerve pathology, while concomitantly accounting for sources of variability that are unrelated to neural dysfunction, is of pivotal importance in clinical practice (Lanza et al., 2017). However, the declaration of an “abnormal” result critically requires the prior definition of what is “normal” and this definition dictates the sensitivity and specificity of the diagnostic test. Other authors (Di Lazzaro et al., 1999; Rossini et al., 2015) have provided reference values for the conduction latencies to several upper and lower extremity muscles, although these values do not or do only partially account for differences in body height, age, and sex.

Body height has consistently been shown to positively correlate with all MEP latency parameters to leg muscles (Chu, 1989; Claus, 1990; Furby et al., 1992), although a few studies suggested that central motor conduction time (CMCT) is independent of body height (Booth et al., 1991). For MEPs recorded from upper limb muscles, most studies suggest that CMCT is indeed independent of height (Chu, 1989; Claus, 1990; Furby et al., 1992; van der Kamp et al., 1996; Wochnik-Dyjas et al., 1997), but the opposite has been reported as well (Ghezzi et al., 1991). As such, the effect of age on CMCT has been described, but heterogeneously: in some studies age has been weakly associated with increased CMCT to leg muscles (Matsumoto et al., 2012), whereas others have not found a clear relation between age and CMCT (Claus, 1990; van der Kamp et al., 1996). Of note, one study (Mano et al., 1992) even found decreased CMCT to hand muscles in the elderly.

Regarding sex differences of MEP, these have rarely been studied; however, significantly longer peripheral motor conduction times (PMCT) and cortical latencies from the First Dorsal Interosseus muscle (FDI) in males have been reported (Mills and Nithi, 1997; Cantone et al., 2019). Significantly shorter PMCT and MEP cortical latencies in females to both upper and lower extremity muscles have been documented (Matamala et al., 2013). Also, sex differences of MEP parameters between male and female subjects have been described (Chu, 1989), although the influence of significantly different body heights could not be ruled out (Toleikis et al., 1991).

As a limitation, these studies were often based on relatively small sample sizes, which implies a reduced sensitivity for weak correlations between MEP features and individual physical characteristics. Furthermore, several technical and procedural factors make it difficult to obtain normative data and to compare the values established by different laboratories. As an example, although some authors conducted the MEP study in healthy subjects, thus demonstrating a positive correlation between MEP cortical latency and both height and age, the study design did not permit to assess peripheral or central motor conduction times (Säisänen et al., 2008). Additionally, the conducted magnetic resonance imaging (MRI)-based navigated TMS is not readily available to most MEP laboratories and leads to significantly shorter MEP latency measures (Julkunen et al., 2009). Therefore, the reliable identification of normal or abnormal MEPs requires a strictly defined methodology and a comprehensive characterization of MEPs in a large healthy population.

In order to identify those factors that are likely to affect motor responses, a “real-world” TMS study on 587 healthy subjects, stratified for age, height, and sex, has been recently conducted, thus mimicking a “real life” practice setting, useful for diagnostic TMS purposes (Cantone et al., 2019). Globally, the results showed that MEP cortical latency and PMCT at four limbs positively correlated with both age and height. Additionally, at upper limbs, an independent effect of sex on PMCT and MEP cortical latency was observed, with females showing smaller values than males. CMCT correlated with both age (negatively) and height (positively) when analyzed by a single regression; however, with multiple regression analysis, this significance was no more evident due to the correction for multicollinearity within the dataset (Cantone et al., 2019).

Despite this in-depth characterization of MEPs, the analysis previously reported did not allow to derive height- and age-adjusted normal values for clinical practice. Therefore, we here perform a re-analysis of the data already presented (Cantone et al., 2019) to provide new height- and age-adjusted normal values for total (i.e., MEP latency), central (i.e., CMCT), and peripheral conduction time (i.e., PMCT) of MEPs from a large cohort. Tabulated upper limits of the normal (ULN) for application in clinical practice are given in the Supplementary Tables 1, 2, and the article provides regression formulae that can be entered into the software of most clinical neurophysiology devices to recognize abnormal results already during a clinical measurement. No new data has been collected for this study. Additionally, only MEP latency variables were re-analyzed in this study, since the MEP amplitude has been already shown to be independent of age, height, and sex (Cantone et al., 2019).

## 2. Materials and methods

### 2.1. Subjects and assessment

For this study, data from a large previous cohort has been re-analyzed. Accordingly, for a detailed description of the sample population and all the technical procedures performed, we refer to the above-mentioned study (Cantone et al., 2019). Briefly, a total of 587 consecutive subjects (41.1% males), ranging from 18 to 87 years in age and from 145 to 197 cm in height, were retrospectively included from the TMS Lab of the Policlinico University Hospital “G. Rodolico–San Marco” of Catania, Italy. Race and ethnicity were not recorded, but there was no indication that these differed from the general population in southern Italy. According to the inclusion criteria, none of the subjects had motor deficits or a history of central and peripheral motor or neuromuscular disorders, based on a preliminary interview, a specific medical questionnaire, and a full neurological examination. All subjects had normal mobility and were able to engage every task of daily life without assistance, even the most elderly. Any CNS pathology was also ruled out by brain and spinal magnetic resonance imaging. Therefore, all participants eventually included were neurologically healthy.

Motor evoked potentials were elicited bilaterally through a circular coil applied over the optimal scalp position (“hot spot”) for the contralateral FDI muscle and Tibialis Anterior (TA) muscle, and responses were recorded from these muscles during mild tonic contraction using standard surface EMG electrodes. For the FDI muscle, electrodes were placed over the mid-point of the belly of the muscle, with the reference electrode at the metacarpal-phalangeal joint of the index finger and ground electrode at the radial surface of the wrist. For the TA muscle, the recording electrode was placed at the mid-point of the muscle belly and the reference electrode 3–4 cm distally over the muscle tendon, with the ground electrode over the patella. Tonic muscle contraction was targeted to about 10–20% of the subject’s maximum voluntary contraction force and controlled for by a strain gauge and audio-visual feedback of the recorded surface EMG (Fritz et al., 1997).

Motor evoked potential cortical latency was calculated as the time interval from the TMS artifact to the first negative deflection of the muscular response with respect to the EMG baseline (Rossini et al., 2015). At least five trials were recorded to confirm the reproducibility of the responses (Rossini and Caramia, 1992). The MEP with the shortest latency was considered for CMCT calculation, according to the international guidelines (Rossini et al., 2015; Vucic et al., 2023).

Peripheral motor conduction time was determined in all subjects by peripheral stimulation of the spinal motor roots. For this purpose, the center of the coil was placed dorsally above the 7th cervical (for upper limbs) or the 4th lumbar (for lower limbs) spinous process. PMCT was calculated as the time interval from the TMS artifact to the first negative spike with respect to the EMG baseline. To ensure reliability, at least two reproducible responses were recorded and averaged (Rossini et al., 2015).

**FIGURE 1:**
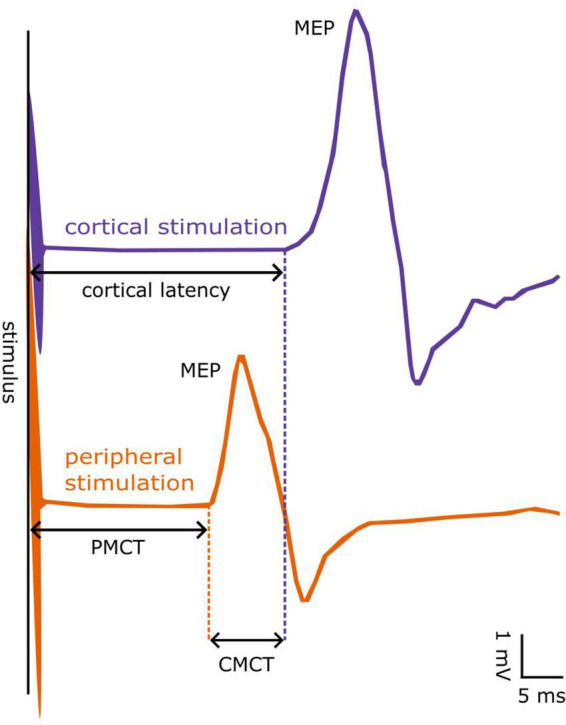
Example of a motor evoked potential (MEP) from the First Dorsal Interosseous muscle. MEPs can be evoked by cortical (blue line) or peripheral stimulation at the spinal cord (orange line). The latency from cortical stimulus to MEP onset is denoted as cortical latency, whereas that obtained from peripheral stimulus to MEP onset as peripheral motor conduction time (PMCT). The “central motor conduction time” (CMCT) is calculated as the difference between cortical latency and PMCT.

Central motor conduction time was defined as the conduction time from motor cortical neurons to the spinal motor output, thus reflecting the conduction of excitation along the cortico-spinal tract. Namely, CMCT was approximated by subtracting the cervical or lumbar PMCT from the shortest MEP cortical latency (Rossini et al., 1985a,b, 1987, 1994; Ugawa et al., 1994), as follows: CMCT = MEP – PMCT. All measured latency parameters (PMCT, MEP cortical latency, and CMCT) are depicted in Figure 1 for visual reference.

As stated, MEP cortical latency was measured during slight tonic contraction of the target muscle, thereby giving the shortest latency from the cortex to the muscle and the shortest CMCT. It should be noted, however, that magnetic stimulation of spinal nerves is effective at the exit of the spinal nerve off the intervertebral foramen (Matsumoto et al., 2013). Thus, using the described methodology, the intradural portion of the spinal nerve (including the cauda equina for lumbar stimulation) is subsumed in the central part of the MEP conduction pathway.

Once collected, data were stored on a dedicated PC by means of an *ad hoc* software that allows to acquire, process, and analyses data (Faro et al., 2010). This custom software is both a hardware-interface to the magnetic stimulation device and data acquisition unit, allowing for standardized collection and data processing of various MEP paradigms from multiple subjects and a database to store and export the obtained data together with the information about the study paradigm.

The study was approved by the Ethics Committee of the Policlinico University Hospital “G. Rodolico – San Marco” of Catania, Italy. All subjects gave written informed consent in accordance with the Declaration of Helsinki of 1964 and its later amendments.

### 2.2. Statistical analysis

All statistical analyses were done in the Python programming language (ver. 3.10) using additional packages numpy (1.23.5), scipy (1.9.3), and matplotlib (3.6.2) for visualization of the results. Multiple regression analysis was conducted, with PMCT, MEP cortical latency, and CMCT as response variables. Additional models were fitted for the absolute side-differences of these parameters. Height, age, and sex were included as independent parameters, with female sex coded as “0” and male sex coded as “1.”

For each of the studied variables, 11 regression models of increasing complexity were fitted: (i) an intercept-only model, (ii) a model with an intercept term and height as a regressor, (iii) a model with an intercept term and age as a regressor, and (iv) a model with an intercept term and both height and age as regressors. The remaining models added male sex as independent parameter: (v) intercept + male, (vi) intercept + male + height, (vii) intercept + male + age, (viii) intercept + male + height + age. Finally, models 9–11 included interaction terms between male sex and height and/or age: (ix) intercept + male + height + male*height, (x) intercept + male + age + male*age, and (xi) intercept + male + height + age + male*height + male*age. Simple regression terms for male sex allow to model that males have an increased (or decreased) MEP latency parameter as compared to females, while interaction terms allow for different effects of height and age in both sexes.

For model selection, Bayesian information criterion (BIC) was calculated for each of these models (Schwarz, 1978), and the model with lowest BIC value was judged as most predictive. If the true model is in a candidate set of models, then, asymptotically, the true model will be selected by the BIC criterion. Also, the BIC is less prone to select models with irrelevant despite significant regressors when compared to the alternative Aikake information criterion (Vrieze, 2012).

For the side-differences of PMCT, MEP cortical latency, and CMCT, models were fitted for the absolute value of the side differences. Note that these absolute values are distributed according to a half-normal distribution, and that the standard deviation (SD) of the side-differences can subsequently be obtained by multiplying the regression result with $\sqrt{\left(0.5\,\pi \right)}$ (Altman, 1993).

Upper limits of the normal (ULN) were calculated for each parameter, adapted for height, age and sex, where warranted. For MEP cortical latency, PMCT, and CMCT, 2.5 × SD (standard deviation) was chosen as cut-off value, such that (assuming normally distributed data) about 0.5% of normal values would be rated as pathological. We chose this conservative cut-off value in favor of high specificity of the obtained ULNs. For the side-differences of these parameters, only the magnitude of the difference was assessed. Consequently, 2.8 × SD was chosen as cut-off value for the ULNs of the side differences, such that (assuming a half-normal distribution) about 0.5% of normal values would be rated as pathological.

Additionally, ULNs were calculated by estimating the 99%-prediction interval using a non-parametric bootstrap method which is robust to deviations from normality of the residuals of the regression (Davison and Hinkley, 1997). If the ULNs obtained from the parametric analysis deviated systematically from the ULNs obtained by bootstrap analysis (indicating a significant effect of non-normality), the model with the 2nd largest BIC was chosen (see Supplementary Figure 5 for an example).

## 3. Results

None of the participants complained of adverse events or undesirable effects during or after TMS. The results of the multiple regression analysis are summarized in Table 1, and the obtained ULNs for all MEP parameters (dependent on height and age, where warranted) are tabulated in Supplementary Tables 1, 2. The BIC values, underlying the selection of the chosen model for each parameter, are tabulated in Supplementary Table 3. Additionally, Supplementary Figures 1–4, available alongside the online version of this article, illustrate all the selected regression models.

**TABLE 1:** Coefficients and corresponding *p-*values for the selected model of all parameters.

	Intercept	*P*-value	Male	*P*-value	Height	*P*-value	Age	*P*-value	SD
**First Dorsal Interosseus muscle**									
PMCT (ms)	2.145	1.8E-03	0.794	3.9E-26	0.059	2.2E-44	0.028	1.2E-43	0.954
PMCT (side diff)	0.486	1.6E-116							0.403
Cortical latency (ms)	6.217	2.2E-14	0.682	3.9E-15	0.072	3.2E-47	0.024	1.9E-24	1.115
Cortical latency (side diff)	0.531	1.9E-112							0.452
CMCT (ms)	4.707	2.3E-20			0.009	3.0E-03	-0.005	2.9E-03	0.873
CMCT (side diff)	0.599	1.8E-131							0.451
**Tibialis Anterior muscle**									
PMCT (ms)	0.206	7.8E-01			0.067	3.9E-52	0.027	3.0E-25	1.278
PMCT (side diff)	0.883	2.0E-94							0.856
Cortical latency (ms)	7.748	2.1E-08	0.390	7.8E-03	0.102	4.0E-34	0.037	4.9E-21	1.905
Cortical latency (side diff)	1.331	1.2E-83							1.404
CMCT (ms)	7.363	2.3E-10	0.358	3.6E-03	0.036	2.0E-07	0.011	1.0E-03	1.598
CMCT (side diff)	1.193	2.3E-107							1.051

*P*-values were not corrected for multiple comparison analysis, but model selection was based on a separate evaluation of the Bayesian information criterion. SD denotes the standard deviation of the residuals. Upper and lower limits of the norm can be calculated from the coefficient values. Side diff = side difference.

### 3.1. PMCT

As previously reported (Cantone et al., 2019) PMCT to upper and lower extremities are significantly correlated to both height and age with positive regression coefficients, indicating that these latency values tended to increase in taller and older subjects. Additionally, PMCT to the FDI was about 0.8 ms longer in male subjects than in females, and this effect was not accounted for by the difference in average height.

### 3.2. MEP cortical latency

Motor evoked potential cortical latencies recorded from the FDI and TA muscle significantly positively correlated with both height and age. Additionally, MEP cortical latency was longer for males then for females to both muscles (0.7 ms to the FDI and 0.4 ms to the TA), and this effected could not be explained by the different average height. Inclusion of sex as a predictor of MEP cortical latency was mainly relevant for recordings from the FDI muscle, as indicated by a substantial decrease of the BIC in comparison to the model without sex as a predictor (see Supplementary Table 3). For the TA muscle, this difference was marginal.

### 3.3. CMCT

**FIGURE 2:**
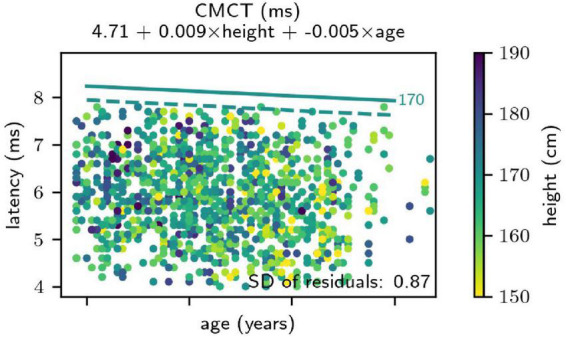
Results of the regression analysis for the central motor conduction time (CMCT) from the First Dorsal Interosseous muscle. The result was independent of sex. Height of the subjects is coded in color. Straight lines indicate the obtained upper limits of the normal (ULN) - from the regression analysis for a height of 170 cm. The solid line was obtained parametrically, relying on the assumption of normally distributed regression residuals, and the dashed line originates from non-parametric bootstrapping. First, note that the parametric ULNs are more conservative than those obtained by bootstrap analysis. Additionally, the analysis unexpectedly revealed a negative correlation between age and CMCT (i.e., a decreasing CMCT at increasing age) and this association was confirmed by the bootstrap analysis.

To the FDI muscle, body height significantly positively correlated with the CMCT. Age, on the other hand, showed a small but significant negative correlation with the CMCT. This negative association was also confirmed using the bootstrap analysis (Figure 2). Sex did not have a significant effect on the CMCT recorded from the FDI muscle. To the TA muscle, also the CMCT significantly positively correlated with both height and age. Additionally, the statistical model including sex as a predictor was slightly better than the “intercept + height + age”-only model. On average, the CMCT to the TA muscle was 0.35 ms longer in male than in female subjects, and this difference was not explained by the difference in height or age.

### 3.4. Side-differences

For the side-differences of all parameters, intercept-only models were chosen for the calculation of normal values, indicating that the left-to-right difference of all MEP parameters was independent of body height, age, and sex. Model selection by BIC indicated that age might have a relevant effect on the side differences of MEP cortical latency to the TA muscle and CMCT to both muscles (with higher age associated with a decrease of side-difference), although this effect could not be reproduced using the non-parametric bootstrap analysis (for an example, see Supplementary Figure 5). As a consequence, the model with the second largest BIC had been chosen, eventually selecting the intercept-only model for the side-differences of all parameters.

### 3.5. Calculation of normal values

For clinical reference, ULNs were calculated from the selected regression models using the subsequently provided formulae. The parametric approach (i.e., based on the regression models) was selected over the results of the non-parametric bootstrap analysis to result in regression formulae that can be entered into the software of most clinical neurophysiology devices for an immediate recognition of normal and abnormal results already during a recording. Lower limits of the normal could be calculated equivalently from the regression formulae by subtracting 2.5 × SD (2.8 × SD for the side-differences) from the predicted mean result, but the interpretation of a MEP latency that is “too short” is difficult in clinical practice. However, these values may be of interest for research purposes.

#### 3.5.1. PMCT, MEP cortical latency, and CMCT

The upper limit of a 99% prediction interval of the analyzed MEP latency parameters can be approximated with the following formula (with male sex coded as “1” and female sex as “0”):


ULN=intercept+male⁢_⁢coef×Sex+height⁢_⁢coef

×Height⁢(cm)+age⁢_⁢coef×Age⁢(years)+2.5×SD.

The corresponding coefficients for each MEP parameter are tabulated in Table 1 (with missing table entries indicating a coefficient of 0). It was verified that this simplified formula, due to the large sample size, lead to only irrelevant deviations from the proper formula for calculating prediction intervals, relying on a Student’s *t* distribution and using a correction of the residual SD for “unobserved” samples (Faraway, 2005). Comparing the obtained ULNs to non-parametric bootstrap prediction, intervals depicted in general a good agreement, although the parametric values tended to be slightly too conservative in some cases (Figure 2).

#### 3.5.2. Side differences

Even in case of normal MEP latency parameters, evaluation of side differences may reveal pathologic results that might remain unnoticed in unilateral measurements. Accordingly, the definition of ULNs for the side-differences of MEP latency parameters increased the diagnostic utility of MEP studies.

Side differences were largely found to be unrelated to height, age, and sex with a predicted influence of age for some parameters, which was not reproducible by non-parametric bootstrap testing. Note that the regression models fitted the absolute value of the side-differences with a presumed mean side difference of 0. According to the literature (Altman, 1993), the absolute side-differences are distributed according to a half-normal distribution and the regression result represents the SD of the side differences after multiplication with the factor $\sqrt{\left(0.5\,\pi \right)}$. Consequently, ULNs were calculated according to the following formula:


$$\mathrm{ULN}=2.8\times \sqrt{\left(0.5\,\pi \right)}\,\mathrm{intercept}$$

with the intercept term tabulated in Table 1 and the factor 2.8 (instead of 2.5) accounting for the assessment of absolute side differences which follow a half-normal distribution instead of a normal distribution. The distribution of side differences of MEP latency parameters was heavy-tailed in comparison to a normal distribution, leading to slightly too liberal ULNs when compared to the non-parametric bootstrap analysis (data not shown). In comparison to previously published ULNs of side differences (Di Lazzaro et al., 1999; Wassermann et al., 2008; Rossini et al., 2015), however, the obtained ULNs were already rather high. Therefore, the 2.8 × SD criterion was accepted for the ULNs of the side-differences.

## 4. Discussion

### 4.1. Main findings

Normal range of TMS measures were previously described (Chu, 1989; Booth et al., 1991; Ghezzi et al., 1991; Furby et al., 1992; van der Kamp et al., 1996; Mills and Nithi, 1997) and gathered (Di Lazzaro et al., 1999; Wassermann et al., 2008; Rossini et al., 2015) although with partially spared and varying results, mainly due to relatively small sample sizes and methodological variations, such as different types of coils (e.g., circular *vs.* figure-of-eight coil), different stimulation approaches (e.g., magnetic spinal root stimulation *vs.* determination of F-wave latencies), and recording from different muscles (at upper and/or lower extremities). Similarly, only few studies took into account the effect of age, sex, and height on MEPs, with controversial results (Matamala et al., 2013).

In this study, data from a large cohort of 587 subjects, recorded with a defined and common methodology (Cantone et al., 2019), was used to overcome the limitations of the previous reports. The present cohort spanned both sexes and a large range of heights and ages, from young adults (18 years) to the elderly (87 years). Nervous system disease was strictly excluded by full medical history, clinical neurologic examination, and MRI scans of the brain and whole spine.

The presented multiple regression analysis thus quantified the effects of height, age, and sex on all latency values (i.e., MEP cortical latency, CMCT, and PMCT). In summary, the following results were obtained: (i) height and age positively correlated with PMCT and MEP cortical latency to both upper and lower extremities; (ii) CMCT to the TA muscle positively correlated with height and age; (iii) CMCT to the FDI muscle positively correlated with height. Additionally, there was a slight negative correlation with age; (iv) sex was a relevant parameter for MEP latency measures, affecting mainly the PMCT and cortical latency to the FDI muscle and, to a lesser degree, cortical latency and CMCT to the TA muscle; (v) side-differences were found to be independent of height, age, or sex.

Regarding the relationship between PMCT and height, this may be explained, at least in part, by the length of peripheral motor nerves along the arms or the legs and the whole subject’s height. Additionally, it is known that aging-related processes are associated with a subclinical functional nerve damage, that eventually leads to a progressive axonal and myeline loss (Vital et al., 1990). As a consequence, negative correlation between progressive age and nerve conduction parameters is a well-known phenomenon (Stålberg and Falck, 1993; Puksa et al., 2003).

For CMCT, although the association with age and height is weaker, the performed regression analysis suggested a positive correlation between height and CMCT recorded from the TA muscle as well. The relationship between CMCT and height in lower limbs is in agreement with several previous studies (Rossini et al., 1987, 2015; Chu, 1989; Claus, 1990; Ghezzi et al., 1991; Ravnborg and Dahl, 1991; Toleikis et al., 1991; Furby et al., 1992; Wochnik-Dyjas et al., 1997; Groppa et al., 2012; Udupa and Chen, 2013). Additionally, a positive correlation between height and all MEP latency values appears reasonable since both length of the limbs (Jarzem and Gledhill, 1993; Fredriks et al., 2005) and of the spine (Zyoud et al., 2020) positively correlate with total body height.

The finding of a positive correlation between body height and CMCT recorded from the FDI muscle, however, innovates the traditional concept that CMCT to upper limb muscles does not correlate with height (Chu, 1989; Claus, 1990; Furby et al., 1992). Additionally, CMCT recorded from the FDI muscle was found inversely correlated with age, thus implying a decrease of CMCT during aging. A similar observation has been made recording MEP from relaxed hand muscles (but not during tonic contraction) in 26 elderly female subjects against a younger control group; this difference was attributed to a pre-parkinsonian stage in the healthy elderly (Mano et al., 1992; Zhang et al., 2021). Indeed, aging has been associated with decreased transcallosal inhibition (Davidson and Tremblay, 2013), and increased cortical excitability in the early stage of Alzheimer’s disease (Vucic and Kiernan, 2017; Vucic et al., 2023) and other neurodegenerative disorders may already be found in clinically unaffected elderly individuals (Lanza et al., 2023). However, it should be stated that the relation between height and CMCT to the upper limbs and both height and age would have left unnoticed in case of a smaller sample size, as in the majority of previous studies.

In this context, the central motor pathways giving rise to MEPs, although known for a long time, continue to be actively investigated, and some aspects of their physiology are still a matter of debate. The time that is needed for the excitatory volley generated within the M1 to reach the spinal cord (referred to as CMCT) includes three components: the time within the cortex, the time along the cortico-spinal tract, and the so-called intraspinal delay (Udupa and Chen, 2013). Height and age may affect TMS variables at any of these levels, although this decoding remains subject of invasive recording techniques. In this study, the exact contribution of central conduction pathways and peripheral nerve conduction to CMCT remain to be elucidated. With the stimulation technique applied here, the CMCT subsumes both the “true” central conduction time and the conduction time in the intradural segment of the spinal nerve until its exit off the intervertebral foramen. At lower limbs, this intradural segment of the nerve root is of significant length and the conduction time between M1 and spinal cord, the time within the spinal cord, and the time within the intradural portion of the nerve root contribute differently to the CMCT recorded from upper and lower limb muscles, which also affects the interpretation of the obtained regression coefficients for height, age, and sex.

Our data also considered the inter-side difference of CMCT to arm and limb muscles, thus adding diagnostic accuracy to routine TMS procedures by allowing to detect and quantify a lateralized prolongation of CMCT, even when this is still within the raw normal values. Of note, side-differences of the latency parameters were independent of height and age.

Lastly, converging evidences support the hypothesis that sex is an important, though often neglected, factor in clinical neurophysiology, which might possibly influence the results of MEPs and the effects of non-invasive brain stimulation techniques. This may be the case of some sex-related brain structural and functional differences, such as total brain volumes, cortical asymmetries, and laterality (Hanlon and McCalley, 2022). Accordingly, data on differences of sex developmental across the life span support the concept of a “sex-guided” cortico-spinal tract maturation. For instance, differences and lateralization in structure-based segments of the cortico-spinal tract were found in healthy term infants during early post-natal period (Saadani-Makki et al., 2019). In early adolescence, female motor tracts seem to reflect more widespread changes, while males may undergo relatively more microstructural changes in projective and associative fibers (Hervé et al., 2009; Bava et al., 2011; Pangelinan et al., 2016). Histopathologically, an elegant research study showed that in the human lateral pyramidal tract at the cervical level (C5), large-size myelinated axons are more dominant in number in males, while small-size myelinated axons are found in relatively lager numbers in females, thus possibly suggesting a sex-difference in the transmission of the responses along this tract (Souma et al., 2008). In line with this finding, neurophysiological nomograms differentiated by sex would be needed to detect even subtle changes.

Altogether, these analyses allowed to draw nomograms according to sex, age, and height and to calculate valid age- and height-adjusted ULNs for all MEP latency parameters. As such, these data will be helpful to minimize the inter-trial and inter-subject variability, thus rendering both clinical and research studies more accurate, more insightful, and of greater translational value, e.g., when it is necessary to elicit corticomotor responses for diagnostic purposes or to assess corticospinal excitability for research studies. Eventually, this will allow to implement a handout of value both for routine daily examinations and for building experimental protocols (Bella et al., 2011a,b; Pennisi et al., 2014; Rossini et al., 2015).

Clinical and research implications would be of particular relevance for some neurological disorders, including ALS, MS, multiple system atrophy (MSA), progressive supranuclear palsy (PSP), and CSM, as previously demonstrated by large or seminal studies. For instance, in MS patients with pyramidal signs in the upper limbs, CMCT is almost always prollonged but, interestingly, in some cases this prolongation occurs despite normal strength in muscles (Bella et al., 2011a,b; Pennisi et al., 2014; Rossini et al., 2015). In ALS, MEPs are a reliable marker of subclinical upper motor neuron damage, particularly among those with prevalent lower motor neuron phenotype/presentation, thus ensuring an early diagnosis in ∼70% of such cases (Zoccolella et al., 2020). Additionally, CMCT can be useful to differentiate ALS and MSA, even for those who clinically had similar upper motor neuron signs, presumably because of selective degeneration of different fibers in the motor descending pathways (Shirota et al., 2022). Similarly, CMCT abnormalities are common in PSP patients, even subclinically, and especially in those with a long disease duration, thus supporting the possible occurrence of functional damage to the corticospinal tract and supplementary motor area in PSP (Abbruzzese et al., 1991). Finally, while the association between MEP abnormalities and motor deficit is well established in CSM, isolated pyramidal signs may not be associated with MEP changes, even when considering age, sex, and height as confounding factors (Lanza et al., 2020b). Additionally, CMCT duration seems to be a useful predictor of the outcome after surgical treatment and early surgery for CSM may produce a beneficial effect on spinal cord functionality that can be reliably detected by MEPs (Takahashi et al., 2008; Capone et al., 2013).

### 4.2. Limitations

As per our previous report (Cantone et al., 2019), some limitations should be acknowledged: (i) as for all retrospective studies, a selection bias cannot be entirely excluded, although the subjects were carefully screened; (ii) an estimation of the peripheral nerve conduction velocity would have been useful to rule out a peripheral nervous system disease, although this goes beyond a routine TMS exam; (iii) limb length was not measured; (iv) as mentioned, PMCT was estimated by spinal root stimulation, which also affects the estimation of the CMCT. As a consequence, the effects of height, age, and sex on CMCT, especially to lower limbs, might have been overestimated in this study. The obtained results could be verified by calculation of the CMCT using the F waves from the recorded muscles, although this analysis cannot be routinely performed in every lab; (v) The timing of testing during the menstrual cycle and its potential effect on MEPs was not considered (Smith et al., 1999, 2002), although a conclusive remark on the relationship between TMS and hormonal status has not been firmly established; (vi) neither the original study (Cantone et al., 2019) nor this re-analysis considered morphological changes of the MEP curves that may convey information about underlying nervous system pathology (Nguyen et al., 2019).

### 4.3. Conclusion

In conclusion, for both clinical and research examination, cut-off values able to separate normal and abnormal measurements should be available in every laboratory, for each muscle, and adjusted for age, height, and sex. A right-left comparison is also recommended to detect subtle abnormality on one side. Although often difficult to do in daily clinical practice, one should keep in mind that sensitivity and specificity of measurements may be insufficient if this is not done.

## Data availability statement

The raw data supporting the conclusions of this article will be made available by the authors, without undue reservation.

## Ethics statement

The studies involving human participants were reviewed and approved by the Ethics Committee of the Policlinico University Hospital “G. Rodolico-San Marco” of Catania, Italy. The patients/participants provided their written informed consent to participate in this study.

## Author contributions

MC, GL, GW, and MP contributed to the conception and design of the study. FF, RB, and RF organized the database. GW and RF performed the statistical analysis. MC, GL, and GP wrote the first draft of the manuscript. FF, RB, GP, and MP wrote sections of the manuscript. All authors contributed to manuscript revision, read, and approved the submitted version.
