# Supplementary material for: Sex-specific reference values for total, central, and peripheral latency of motor evoked potentials from a large cohort
Source: Front Hum Neurosci. 2023 Jun 9;17:1152204. doi: 10.3389/fnhum.2023.1152204 (PMC10288153; doi:10.3389/fnhum.2023.1152204)
Supplement: Supplementary file 1 [file Data_Sheet_1.pdf]

Supplementary Material

Sex-specific reference values for total, central, and peripheral latency of motor evoked potentials from a large cohort

Mariagiovanna Cantone, Giuseppe Lanza*, Francesco Fisicaro, Rita Bella, Raffaele Ferri, Giovanni Pennisi, Gunnar Waterstraat, Manuela Pennisi

*** Correspondence:** giuseppe.lanza1@unict.it

Regression results 2

Tabulated upper limits of the normal for the First Dorsal Interosseus (FDI) muscle 2

Tabulated upper limits of the normal for the Tibialis Anterior (TA) muscle 3

Regression result graphic for the First Dorsal Interosseus (FDI) muscle 4

Regression result graphic for the Tibialis Anterior (TA) muscle 6

Model selection 8

BIC values of models 8

Example of a model rejected after bootstrap analysis 9

First Dorsal Interosseus muscle (females)

PMCT							
**Age \ Height**	**150**	**160**	**170**	**180**	**190**	**200**	**LR diff**
**20**	14.0	14.6	15.1	15.7	16.3	16.9	1.7
**30**	14.2	14.8	15.4	16.0	16.6	17.2	1.7
**40**	14.5	15.1	15.7	16.3	16.9	17.5	1.7
**50**	14.8	15.4	16.0	16.6	17.2	17.8	1.7
**60**	15.1	15.7	16.3	16.9	17.5	18.1	1.7
**70**	15.4	16.0	16.6	17.2	17.7	18.3	1.7
**80**	15.7	16.3	16.8	17.4	18.0	18.6	1.7
**90**	15.9	16.5	17.1	17.7	18.3	18.9	1.7
**100**	16.2	16.8	17.4	18.0	18.6	19.2	1.7

Cortical latency							
**Age \ Height**	**150**	**160**	**170**	**180**	**190**	**200**	**LR diff**
**20**	20.2	20.9	21.7	22.4	23.1	23.8	1.9
**30**	20.5	21.2	21.9	22.6	23.3	24.0	1.9
**40**	20.7	21.4	22.1	22.8	23.6	24.3	1.9
**50**	20.9	21.7	22.4	23.1	23.8	24.5	1.9
**60**	21.2	21.9	22.6	23.3	24.0	24.8	1.9
**70**	21.4	22.1	22.8	23.6	24.3	25.0	1.9
**80**	21.7	22.4	23.1	23.8	24.5	25.2	1.9
**90**	21.9	22.6	23.3	24.0	24.8	25.5	1.9
**100**	22.1	22.8	23.6	24.3	25.0	25.7	1.9

CMCT							
**Age \ Height**	**150**	**160**	**170**	**180**	**190**	**200**	**LR diff**
**20**	8.1	8.2	8.2	8.3	8.4	8.5	2.1
**30**	8.0	8.1	8.2	8.3	8.4	8.4	2.1
**40**	8.0	8.1	8.1	8.2	8.3	8.4	2.1
**50**	7.9	8.0	8.1	8.2	8.3	8.3	2.1
**60**	7.9	7.9	8.0	8.1	8.2	8.3	2.1
**70**	7.8	7.9	8.0	8.1	8.2	8.2	2.1
**80**	7.8	7.8	7.9	8.0	8.1	8.2	2.1
**90**	7.7	7.8	7.9	8.0	8.1	8.1	2.1
**100**	7.7	7.7	7.8	7.9	8.0	8.1	2.1

First dorsal interosseus (males)

PMCT							
**Age \ Height**	**150**	**160**	**170**	**180**	**190**	**200**	**LR diff**
**20**	14.8	15.3	15.9	16.5	17.1	17.7	1.7
**30**	15.0	15.6	16.2	16.8	17.4	18.0	1.7
**40**	15.3	15.9	16.5	17.1	17.7	18.3	1.7
**50**	15.6	16.2	16.8	17.4	18.0	18.6	1.7
**60**	15.9	16.5	17.1	17.7	18.3	18.8	1.7
**70**	16.2	16.8	17.4	17.9	18.5	19.1	1.7
**80**	16.5	17.0	17.6	18.2	18.8	19.4	1.7
**90**	16.7	17.3	17.9	18.5	19.1	19.7	1.7
**100**	17.0	17.6	18.2	18.8	19.4	20.0	1.7

Cortical latency							
**Age \ Height**	**150**	**160**	**170**	**180**	**190**	**200**	**LR diff**
**20**	20.9	21.6	22.3	23.0	23.8	24.5	1.9
**30**	21.1	21.9	22.6	23.3	24.0	24.7	1.9
**40**	21.4	22.1	22.8	23.5	24.2	25.0	1.9
**50**	21.6	22.3	23.0	23.8	24.5	25.2	1.9
**60**	21.9	22.6	23.3	24.0	24.7	25.4	1.9
**70**	22.1	22.8	23.5	24.2	25.0	25.7	1.9
**80**	22.3	23.0	23.8	24.5	25.2	25.9	1.9
**90**	22.6	23.3	24.0	24.7	25.4	26.2	1.9
**100**	22.8	23.5	24.2	25.0	25.7	26.4	1.9

CMCT							
**Age \ Height**	**150**	**160**	**170**	**180**	**190**	**200**	**LR diff**
**20**	8.1	8.2	8.2	8.3	8.4	8.5	2.1
**30**	8.0	8.1	8.2	8.3	8.4	8.4	2.1
**40**	8.0	8.1	8.1	8.2	8.3	8.4	2.1
**50**	7.9	8.0	8.1	8.2	8.3	8.3	2.1
**60**	7.9	7.9	8.0	8.1	8.2	8.3	2.1
**70**	7.8	7.9	8.0	8.1	8.2	8.2	2.1
**80**	7.8	7.8	7.9	8.0	8.1	8.2	2.1
**90**	7.7	7.8	7.9	8.0	8.1	8.1	2.1
**100**	7.7	7.7	7.8	7.9	8.0	8.1	2.1

Supplementary Table 1. Obtained upper limits of the normal for the motor evoked potentials (MEPs) from the First Dorsal Interosseus muscle, stratified for height (cm) and age (years); CMCT = central motor conduction time (ms); PMCT = peripheral motor conduction time (ms); LR diff = left-right difference (ms).

frontiers

Tibialis Anterior muscle (females)

PMCT							
Age \ Height	150	160	170	180	190	200	LR diff
20	14.1	14.7	15.4	16.1	16.7	17.4	3.1
30	14.3	15.0	15.7	16.3	17.0	17.7	3.1
40	14.6	15.3	15.9	16.6	17.3	18.0	3.1
50	14.9	15.5	16.2	16.9	17.6	18.2	3.1
60	15.1	15.8	16.5	17.1	17.8	18.5	3.1
70	15.4	16.1	16.7	17.4	18.1	18.8	3.1
80	15.7	16.3	17.0	17.7	18.4	19.0	3.1
90	15.9	16.6	17.3	18.0	18.6	19.3	3.1
100	16.2	16.9	17.6	18.2	18.9	19.6	3.1

Cortical latency							
Age \ Height	150	160	170	180	190	200	LR diff
20	28.5	29.5	30.6	31.6	32.6	33.6	4.7
30	28.9	29.9	30.9	32.0	33.0	34.0	4.7
40	29.3	30.3	31.3	32.3	33.4	34.4	4.7
50	29.7	30.7	31.7	32.7	33.7	34.7	4.7
60	30.0	31.0	32.1	33.1	34.1	35.1	4.7
70	30.4	31.4	32.4	33.5	34.5	35.5	4.7
80	30.8	31.8	32.8	33.8	34.9	35.9	4.7
90	31.2	32.2	33.2	34.2	35.2	36.2	4.7
100	31.5	32.5	33.6	34.6	35.6	36.6	4.7

CMCT							
Age \ Height	150	160	170	180	190	200	LR diff
20	16.9	17.3	17.6	18.0	18.3	18.7	4.2
30	17.0	17.4	17.7	18.1	18.4	18.8	4.2
40	17.1	17.5	17.8	18.2	18.5	18.9	4.2
50	17.2	17.6	17.9	18.3	18.6	19.0	4.2
60	17.3	17.7	18.0	18.4	18.8	19.1	4.2
70	17.4	17.8	18.1	18.5	18.9	19.2	4.2
80	17.5	17.9	18.3	18.6	19.0	19.3	4.2
90	17.7	18.0	18.4	18.7	19.1	19.4	4.2
100	17.8	18.1	18.5	18.8	19.2	19.5	4.2

Tibialis Anterior muscle (males)

PMCT							
Age \ Height	150	160	170	180	190	200	LR diff
20	14.1	14.7	15.4	16.1	16.7	17.4	3.1
30	14.3	15.0	15.7	16.3	17.0	17.7	3.1
40	14.6	15.3	15.9	16.6	17.3	18.0	3.1
50	14.9	15.5	16.2	16.9	17.6	18.2	3.1
60	15.1	15.8	16.5	17.1	17.8	18.5	3.1
70	15.4	16.1	16.7	17.4	18.1	18.8	3.1
80	15.7	16.3	17.0	17.7	18.4	19.0	3.1
90	15.9	16.6	17.3	18.0	18.6	19.3	3.1
100	16.2	16.9	17.6	18.2	18.9	19.6	3.1

Cortical latency							
Age \ Height	150	160	170	180	190	200	LR diff
20	28.9	29.9	31.0	32.0	33.0	34.0	4.7
30	29.3	30.3	31.3	32.4	33.4	34.4	4.7
40	29.7	30.7	31.7	32.7	33.7	34.8	4.7
50	30.0	31.1	32.1	33.1	34.1	35.1	4.7
60	30.4	31.4	32.5	33.5	34.5	35.5	4.7
70	30.8	31.8	32.8	33.8	34.9	35.9	4.7
80	31.2	32.2	33.2	34.2	35.2	36.3	4.7
90	31.5	32.6	33.6	34.6	35.6	36.6	4.7
100	31.9	32.9	34.0	35.0	36.0	37.0	4.7

CMCT							
Age \ Height	150	160	170	180	190	200	LR diff
20	17.3	17.6	18.0	18.3	18.7	19.0	4.2
30	17.4	17.7	18.1	18.4	18.8	19.1	4.2
40	17.5	17.8	18.2	18.5	18.9	19.2	4.2
50	17.6	17.9	18.3	18.6	19.0	19.4	4.2
60	17.7	18.0	18.4	18.8	19.1	19.5	4.2
70	17.8	18.2	18.5	18.9	19.2	19.6	4.2
80	17.9	18.3	18.6	19.0	19.3	19.7	4.2
90	18.0	18.4	18.7	19.1	19.4	19.8	4.2
100	18.1	18.5	18.8	19.2	19.5	19.9	4.2

Supplementary Table 2. Obtained upper limits of the normal for motor evoked potentials (MEPs) from the Tibialis Anterior muscle, stratified for height (cm) and age (years); CMCT = central motor conduction time (ms); PMCT = peripheral motor conduction time (ms); LR diff = left-right difference (ms).

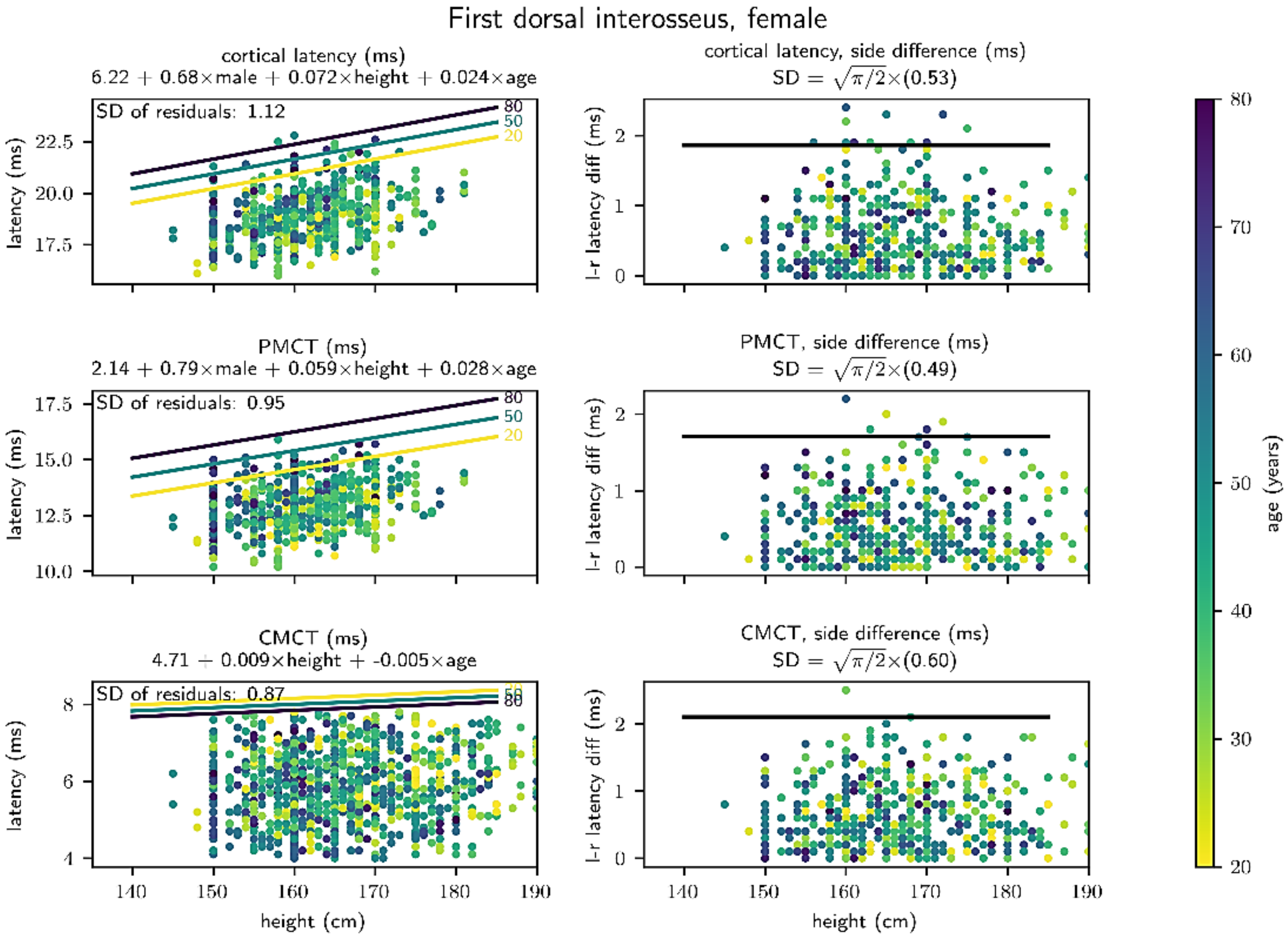

Supplementary Figure 1. Results of the regression analysis for MEP responses from the First Dorsal Interosseous muscle in females. Age of the subjects is coded in color. Straight lines indicate the obtained upper limits of the normal (ULN) from the regression analysis, with age coded in color, where appropriate. The colored numbers “30”, “50”, and “80” (where present) denote the age for which the ULN line of the same color had been drawn. The models for side-differences fitted the standard deviation (SD) of the side differences with a fixed mean of 0. MEP = motor evoked potential; CMCT = central motor conduction time (ms); PMCT = peripheral motor conduction time (ms).

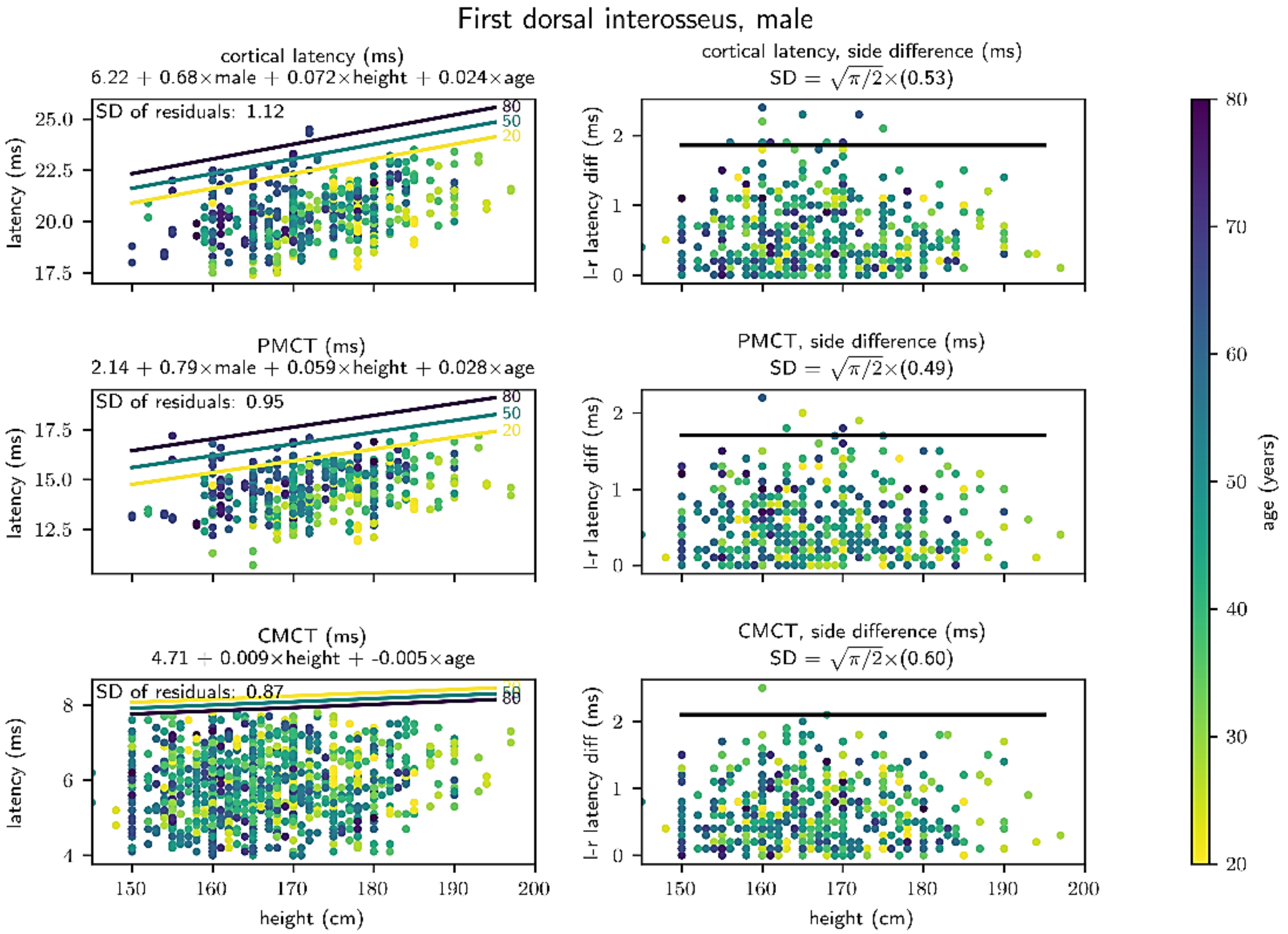

Supplementary Figure 2. Results of the regression analysis for MEP responses from the First Dorsal Interosseous muscle in males. Refer to supplementary figure 1 for details.

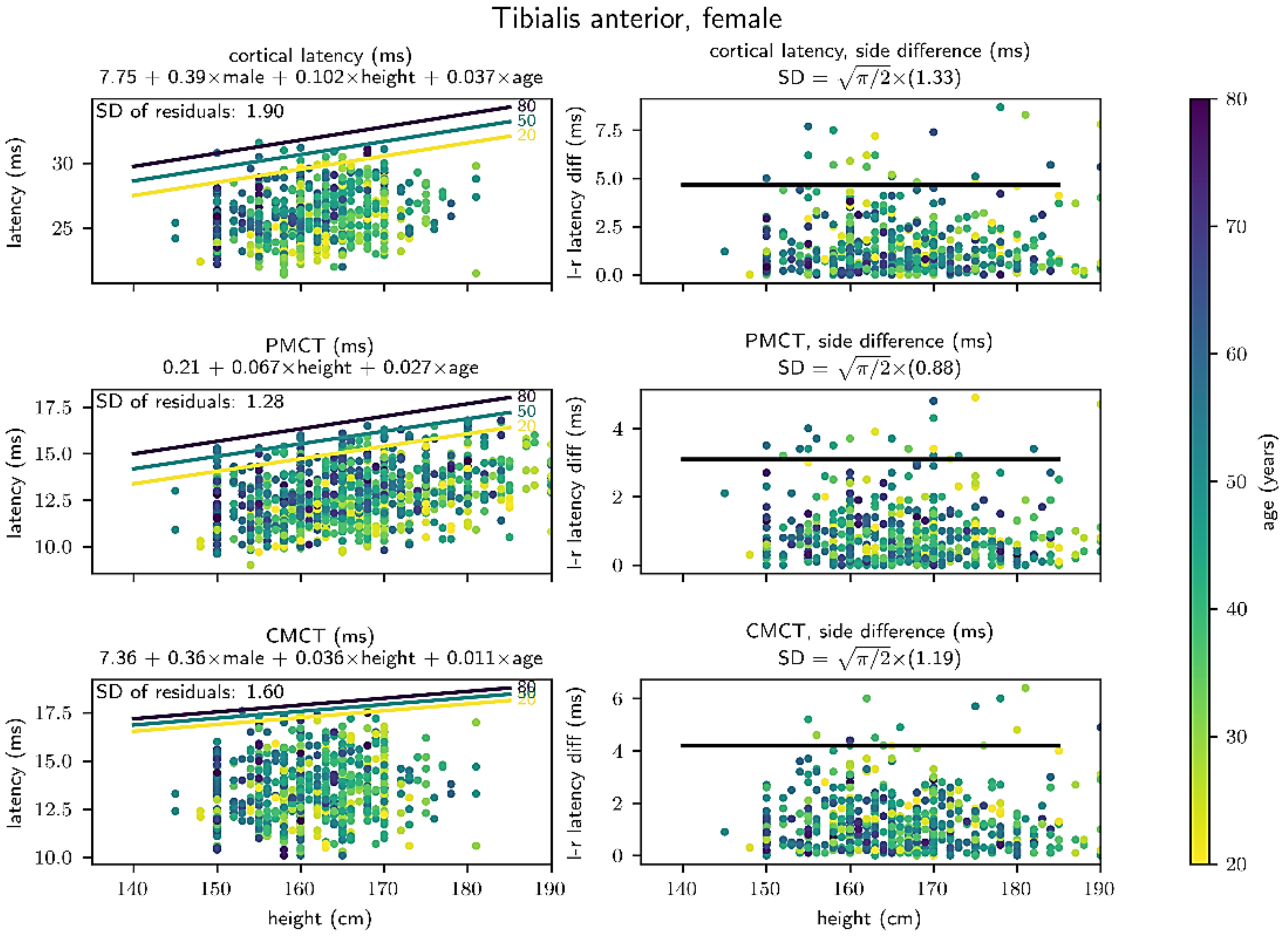

Supplementary Figure 3. Results of the regression analysis for MEP responses from the Tibialis Anterior muscle in females. Refer to supplementary figure 1 for details.

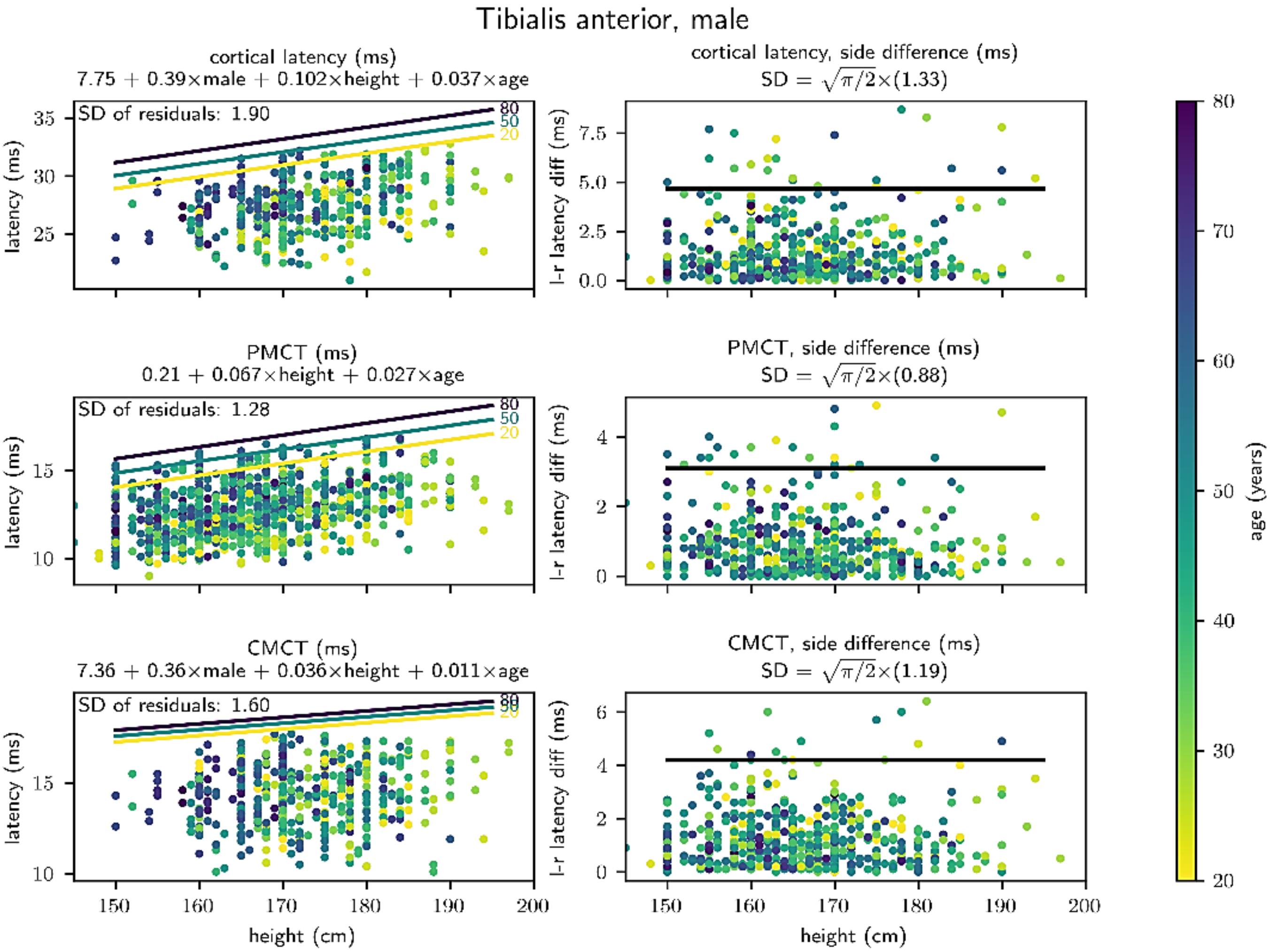

Supplementary Figure 4. Results of the regression analysis for MEP responses from the Tibialis anterior muscle in males. Refer to supplementary figure 1 for details.

First Dorsal Interosseus muscle

	PMCT	PMCT (side difference)	Cortical latency	Cortical latency (side difference)	CMCT	CMCT (side difference)
**Intercept only**	643.9	**-1061.4**	875.1	**-925.9**	-291.4	-928.0
**Height**	275.6	-1056.5	481.7	-920.7	-297.2	-923.6
**Age**	559.9	-1055.6	841.9	-919.8	-297.2	**-928.4**
**Height + age**	23.0	-1051.1	339.4	-914.4	**-299.0**	-922.9
**Male**	189.6	-1055.9	510.4	-919.6	-284.4	-921.8
**Male + height**	103.3	-1050.2	381.9	-915.2	-295.8	-919.8
**Male + age**	106.8	-1050.0	486.5	-913.4	-290.5	-922.0
**Male + height + age**	**-82.2**	-1044.7	**284.5**	-908.8	-294.7	-917.5
**Male + height + male * height**	110.4	-1044.1	388.9	-913.0	-288.9	-913.7
**Male + age + male * age**	113.3	-1045.0	491.5	-907.1	-284.6	-915.8
**Male + height + age + male * height + male * age**	-69.5	-1033.1	298.6	-900.3	-281.9	-905.3

Tibialis Anterior muscle

	PMCT	PMCT (side difference)	Cortical latency	Cortical latency (side difference)	CMCT	CMCT (side difference)
**Intercept only**	863.5	**-176.8**	1862.8	405.1	1201.9	65.3
**Height**	699.0	-170.9	1642.0	409.8	1140.9	70.9
**Age**	822.5	-170.6	1829.4	**404.1**	1203.1	**61.6**
**Height + age**	**598.0**	-164.6	1541.1	409.8	1130.6	67.9
**Male**	771.7	-170.5	1707.7	411.1	1145.2	71.0
**Male + height**	697.5	-164.6	1622.9	416.1	1132.9	74.4
**Male + age**	738.6	-164.3	1683.0	409.7	1149.2	67.7
**Male + height + age**	605.0	-158.2	**1541.1**	416.0	**1129.1**	73.4
**Male + height + male * height**	704.4	-158.2	1629.9	421.0	1140.0	80.6
**Male + age + male * age**	745.7	-158.2	1689.9	415.8	1156.2	74.1
**Male + height + age + male * height + male * age**	616.3	-145.9	1553.2	426.5	1143.1	86.0

Supplementary Table 3. Bayesian information criterion (BIC) values for all tested models. Models with smallest BIC are highlighted in gray and by bold font. Where indicated by red coloring, the model with 2nd largest BIC was chosen due to strong deviations between bootstrap and parametric prediction intervals (see supplementary figure 5 for an example). Models including sex as a parameter are placed below the black horizontal line.

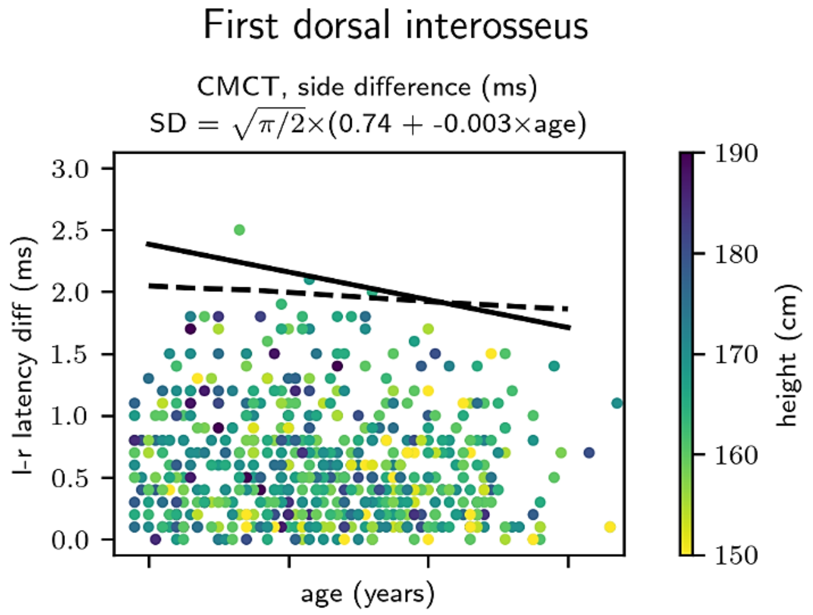

Supplementary Figure 5. Inadequately selected model for the side-difference of CMCT for MEP from the First Dorsal Interosseous muscle. Regression results are shown with age as independent variable (abscissa) and with body height coded in color. In addition to upper limits of the parametric prediction interval (straight line), a prediction interval was obtained by a bootstrap method (dashed line). Model selection by the Bayesian information criterion (BIC) overestimated the effect of age on the side difference of central motor conduction time (CMCT). This is due to non-normal distribution of the predictors and/or response variables. In case of strong systematic deviations between parametric and bootstrap confidence intervals, the model with 2nd largest BIC was chosen (marked in red in supplementary table 3). l-r = left-right; diff = difference.
